# Synthesis and Evaluation of Mannitol-Based Inhibitors for Lipopolysaccharide Biosynthesis

**DOI:** 10.1155/2016/3475235

**Published:** 2016-02-11

**Authors:** Richard E. Johnsson

**Affiliations:** Centre for Analysis and Synthesis, Lund University, P.O. Box 124, 221 00 Lund, Sweden

## Abstract

Antibiotic resistance is a serious threat against humankind and the need for new therapeutics is crucial. Without working antibiotics, diseases that we thought were extinct will come back. In this paper two new mannitol bisphosphate analogs, 1,6-dideoxy-1,6-diphosphoramidate mannitol and 1,6-dideoxy-1,6-dimethansulfonamide mannitol, have been synthesized and evaluated as potential inhibitors of the enzyme GmhB in the biosynthesis of lipopolysaccharides. 1,6-Dideoxy-1,6-diphosphoramidate mannitol showed promising result in computational docking experiments, but neither phosphate analog showed activity in the Kirby-Bauer antibiotic susceptibility test.

## 1. Introduction

Antibiotic resistant microorganisms are among the greatest threats to human health [1–3]. The number of pathogens that acquire resistance to multiple classes of antibiotics is increasing and the need to develop new classes of antibiotics aiming for new targets is fundamental for maintaining the antibiotic era [4, 5]. There are today Gram-negative bacteria, for example,* Acinetobacter baumannii* that are resistant to all FDA approved drugs [6, 7]. Therefore, new molecules with new mechanisms of action are critical for our future. The major component of the outer membrane of Gram-negative bacteria is lipopolysaccharides (LPS), which are made up of a wide range of different carbohydrates. This membrane functions as a protective barrier against antibiotics and antibacterial compounds [7, 8].

**Figure 1:**
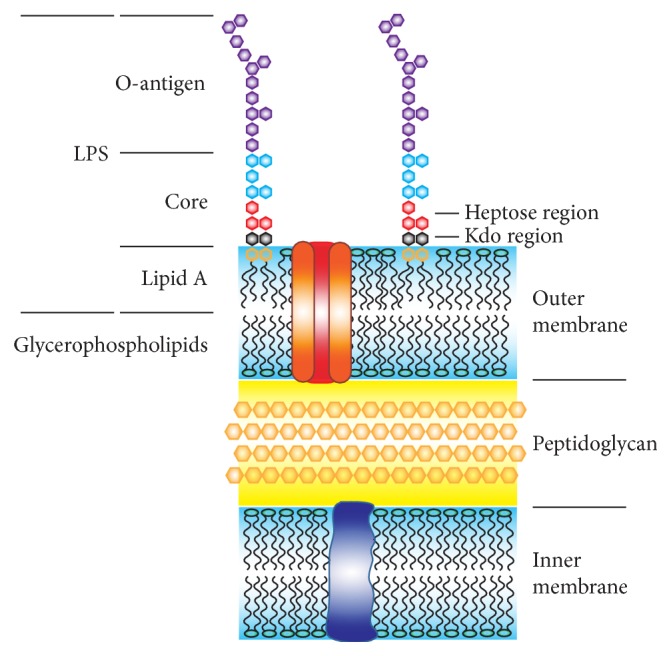
Schematic representation of a Gram-negative bacterial cell envelope (adapted from [10]).

LPS consists of three regions: lipid A, which anchors it to the outer membrane, the core region, and the O-antigen (Figure 1). The core region is usually connected to lipid A with one or two 3-deoxy-d-manno-octulosonic acid (Kdo) residues which are linked to a second carbohydrate, l-glycero-d-manno-heptose (l,d-Hep). The minimal LPS structure required for the growth of* Escherichia coli* consists of lipid A linked to two Kdo units [9]. Gram-negative bacteria without access to heptose produce a heptose-free LPS. This phenotype, called the deep-rough phenotype, is a series of characteristics that collectively reflects changes in the outer membrane leading to its instability, including hypersensitivity to hydrophobic dyes, detergents, and lipophilic antibiotics [10, 11]. Inhibition of the l,d-Hep biosynthesis pathway should hence not influence cell propagation; however, it would result in a truncated LPS that makes the bacteria vulnerable to external stresses, such as the complement system. In this way, the virulence of the bacteria rather than cell growth is targeted and the risk for development of antibiotic resistance may be reduced [12]. In complex cases with immunocompromised hosts, an LPS inhibitor could be administered as an adjuvant making a wide range of available lipophilic antibiotics effective on Gram-negative bacteria as well.

Biosynthesis of l,d-Hep has been completely elucidated in five steps involving four enzymes: GmhA, HldE, GmhB, and HldD [13]. HldE is a bifunctional enzyme that in some species has been replaced by two enzymes, HldA and HldC [14].

**Figure 2:**
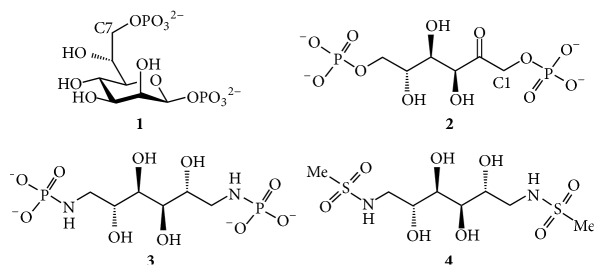
The enzyme GmhB is a dephosphatase that cleaves the phosphate in position C-7 of d-glycero-*β*-d-manno-heptose 1,7-bisphosphate (**1**). The enzyme also shows activity for fructose 1,6-bisphosphate (**2**). Compounds** 3** and** 4** were synthesized as analogs to compound** 2**.

The enzyme GmhB is a phosphatase that catalyzes the removal of the phosphate in position C-7 of d-glycero-*β*-d-manno-heptose 1,7-bisphosphate (**1**) (Figure 2). The protein has also shown fructose 1,6-bisphosphate (**2**) dephosphorylation activity, cleaving the phosphate in position C-1 [15]. Hexitol 1,6-bisphosphate, a mixture of glucitol and mannitol 1,6-bisphosphate, can, for example, inhibit class I and class II fructose 1,6-bisphosphate aldolase [16, 17] and fructose 1,6-bisphosphate phosphatase [18], indicating that the hexitol scaffold can be an effective scaffold to inhibit fructose-binding sites. Mabiala-Bassiloua et al. have recently showed that mannitol 1,6-bisphosphate was a better inhibitor than glucitol 1,6-bisphosphate for rabbit muscle and* Helicobacter pylori* aldolase [19]. To our knowledge, no inhibitors have been made towards GmhB and herein we present the design, synthesis, and evaluation of two different phosphate analogs. It is unknown if fructose 1,6-bisphosphate is a substrate for GmhB in an open linear form or in a furanose configuration and in this study we evaluated 1,6-dideoxy-1,6-diphosphoramidate mannitol (**3**) as a charged phosphate analog and 1,6-dideoxy-1,6-dimethansulfonamide mannitol (**4**) as an uncharged analog to the open linear chain configuration of fructose (Figure 2).

## 2. Materials and Methods

### 2.1. Synthesis Description

*General Experimental Details.* NMR spectra were recorded with a Bruker Avance II 400 MHz and ^1^H NMR spectra were assigned using 2D methods. Chemical shifts are given in ppm downfield from the signal for Me_4_Si, with reference to residual C_6_D_6_ (^1^H NMR 7.16, ^13^C NMR 128.06) or D_2_O (^1^H NMR 4.79). Reactions were monitored by TLC using alumina plates coated with silica gel and visualized either by using UV light or by charring with* para*-anisaldehyde. Preparative chromatography was performed with silica gel (35–70 *μ*m, 60 Å) or Biotage Isolera One SNAP columns. Dichloromethane and toluene were dried on an mBraun solvent dispense system, benzyl alcohol, triethylamine, and phosphorous trichloride were distilled prior to use, and pyridine (extra dry) and all other reagents were used as supplied from the manufacturer. NMR spectra of compounds** 3**,** 4**,** 8**,** 9**, and** 10** are provided in the Supplementary Material available online at http://dx.doi.org/10.1155/2016/3475235.

*1,6-Dideoxy-1,6-diphosphoramidate Mannitol (* ***3*** *).* Compound** 9** (95 mg, 0.09 mmol) was dissolved in EtOAc/EtOH/H_2_O (3 : 5 : 2, 4 mL) and Pd/C (10%, 66 mg) was added and the mixture was hydrogenolysed at atmospheric pressure. After 4 h the mixture was filtered through Celite and concentrated down to approximately 1 mL, H_2_O (20 mL) was added, and the mixture was lyophilized to give** 3** (27 mg, 89%). [*α*]_D_^21^ +19 (c 0.5, D_2_O), ^1^H NMR (D_2_O): *δ* 3.93 (bs, 2H), 3.78 (bs, 2H), 3.40 (bs, 2H), 3.06 (bs, 2H). ^13^C NMR (D_2_O): *δ* 70.5 (CH), 66.9 (CH), 42.5 (CH_2_). ^31^P NMR (D_2_O): *δ* 0.02. HRMS (ESI) calcd. for C_6_H_17_N_2_O_10_P_2_ (M)^−^: 339.0358, found: 339.0382.

*1,6-Dideoxy-1,6-dimethansulfonamide Mannitol (* ***4*** *).* Compound** 10** (55 mg, 0.08 mmol) was dissolved in EtOAc/EtOH/H_2_O (3 : 5 : 2, 3.3 mL) and Pd/C (10%, 100 mg) was added and the mixture was hydrogenolysed at atmospheric pressure. After 3 h Pd/C (10%, 50 mg) was added and the mixture was hydrogenolysed at atmospheric pressure for another 18 h. The mixture was filtered through Celite and concentrated down to approximately 1 mL, H_2_O (20 mL) was added, and the mixture was lyophilized to give** 4** (26 mg, 99%). [*α*]_D_^21^ +21 (c 0.4, D_2_O), ^1^H NMR (D_2_O): *δ* 3.73–3.80 (m, 4H, H-2, H-3, H-4, H-5), 3.47 (dd, 2H,* J* 13.5, 2.2 Hz, H-1, H-6), 3.20 (dd, 2H,* J* 13.5, 6.2 Hz, H-1′, H-6′), 3.09 (s, 6H, SCH_3_). ^13^C NMR (D_2_O): *δ* 69.7, 69.3, 45.7, 38.9. HRMS (ESI) calcd. for C_8_H_20_N_2_O_8_S_2_Na (M+Na)^+^: 359.0559, found: 359.0586.

*1,6-Di-O-trityl Mannitol (* ***6*** *).* Mannitol (**5**) (5.05 g, 27.57 mmol) was coevaporated from pyridine two times and then suspended in pyridine (95 mL) and stirred at r.t. under nitrogen. Trityl chloride (18.55 g, 66.55 mmol) was added followed by AgNO_3_ (12.20 g, 71.84 mmol). After 18 h CH_2_Cl_2_ was added and the mixture was washed twice with NaHCO_3_ (sat. aq.). The aqueous phase was extracted once with CH_2_Cl_2_ and the combined organic phase was dried (MgSO_4_), concentrated, and coevaporated from toluene. The residue was purified by chromatography (Biotage Isolera One, SNAP 50 g, EtOAc 0–100% in heptane; the material was split into four equal parts before chromatography and the clean fractions were pooled) to give** 6** (11.17 g, 61%). Spectral data was in agreement with previously published data [20].

*2,3,4,5-Tetra-O-benzyl Mannitol (* ***7*** *).* Compound** 6** (11.06 g, 16.58 mmol) was dissolved in THF (100 mL) and stirred at r.t. under argon. Benzyl bromide (9 mL, 75.67 mmol) was added followed by NaH (60% suspension in oil, 4.03 g, and 100.75 mmol). After 18 h the mixture was filtered through Celite, eluting with Et_2_O and then washed once with H_2_O, dried (MgSO_4_), and concentrated to give 1,6-di-*O*-trityl-2,3,4,5-tetra-*O*-benzyl mannitol that was used in the next step without further purification. 1,6-Di-*O*-trityl-2,3,4,5-tetra-*O*-benzyl mannitol was dissolved in n-BuOH/CH_2_Cl_2_ (1 : 1, 230 mL) and trifluoroacetic acid (23 mL, 135 mmol) was added and the mixture was stirred at r.t. After 18 h the mixture was poured into NaHCO_3_ (sat. aq., 250 mL) and stirred for 5 min. The phases were separated and the organic phase was concentrated down. The residue was purified by chromatography (CH_2_Cl_2_ followed by pentane/EtOAc 1 : 1) to give** 7** (6.87 g, 76% over two steps). Spectral data was in agreement with previously published data [21].

*1,6-Azido-1,6-dideoxy-2,3,4,5-tetra-O-benzyl Mannitol (* ***8*** *).* Compound** 7** (1.37 g, 2.53 mmol) was dissolved in dry pyridine (4 mL) and tosyl chloride (1.22 g, 6.39 mmol) was added. The mixture was stirred for 18 h at r.t. under N_2_. H_2_O was added and the mixture was extracted twice with CH_2_Cl_2_ and the combined organic phase was washed twice with H_2_O, dried (MgSO_4_), concentrated, and coevaporated from toluene twice to give 2,3,4,5-tetra-*O*-benzyl-1,2-tosyl mannitol. This material was dissolved in DMSO (3 mL) and NaN_3_ (650 mg, 10.0 mmol) was added. The mixture was heated to 100°C for 18 h followed by 150°C for another 18 h. The reaction was allowed to cool before brine was added. The mixture was extracted thrice with ether, dried (MgSO_4_), concentrated, and coevaporated from toluene. The residue was purified by chromatography (Biotage Isolera One, SNAP 50 g, EtOAc 5–95% in heptane) to give** 8** (774 mg, 60%) as a yellow oil. IR (ATR) *υ* 2096 cm^−1^ (N_3_), [*α*]_D_^24^ +100 (c 0.5, Benzene), ^1^H NMR (C_6_D_6_): *δ* 7.26–7.29 (m, 8H, ArH), 7.14–7.19 (m, 8H, ArH), 7.06–7.12 (m, 4H, ArH), 4.64, 4.49 (ABq, 2H each,* J* 11.5 Hz, PhCH_2_), 4.38, 4.18 (ABq, 2H each,* J* 11.5 Hz, PhCH_2_), 3.87–3.89 (m, 2H, H-3, H-4), 3.64–3.68 (m, 2H, H-2, H-5), 3.37, 3.25 (dABq, 2H each,* J* 13.4, 5.3, 2.8 Hz, H-1, H-1′, H-6, H-6′). ^13^C NMR (C_6_D_6_): *δ* 138.8, 138.2, 128.2, 128.1, 128.00, 127.96, 79.9, 79.2, 74.6, 72.2, 50.9. HRMS (ESI) calcd. for C_34_H_36_N_6_O_4_Na (M+Na)^+^: 615.2696, found: 615.2687.

*1,6-Dideoxy-1,6-di-(di-benzyl-phosphoramidate)-2,3,4,5-tetra-O-benzyl Mannitol (* ***9*** *).* Compound** 8** (94 mg, 0.16 mmol) was dissolved in dry toluene (4 mL) and stirred at r.t. under N_2_. Tribenzyl phosphite [22] (560 mg, 1.59 mmol) dissolved in dry toluene (2 mL) was added and the mixture was heated to 90°C. After 18 h the mixture was allowed to cool down and then concentrated. The residue was purified by chromatography (SiO_2_, heptane/EtOAc 1 : 4) to give** 9** (103 mg, 61%) as a clear oil. [*α*]_D_^21^ +19 (c 0.6, C_6_D_6_), ^1^H NMR (C_6_D_6_): *δ* 6.96–7.40 (m, 40H, ArH), 5.00 (d, 4H,* J* 7.8 Hz, PhCH_2_), 4.97 (d, 4H,* J* 7.9 Hz, PhCH_2_), 4.79, 4.71 (ABq, 2H each,* J* 11.3 Hz, PhCH_2_), 4.46, 4.39 (ABq, 2H each,* J* 11.6 Hz, PhCH_2_), 4.00–4.04 (m, 2H, H-3, H-4), 3.82–3.87 (m, 2H, H-2, H-5), 3.38–3.47 (m, 4H, H-1, H-1′, H-6, H-6′), 3.28–3.37 (m, 2H, NH). ^13^C NMR (C_6_D_6_): *δ* 139.4, 138.9, 137.5, 137.4, 128.7, 128.2, 127.94, 127.91, 127.7, 80.6, 80.5, 79.5, 74.7, 72.0, 68.2, 68.1, 41.6. ^31^P NMR (C_6_D_6_): *δ* 23.9. HRMS (ESI) calcd. for C_62_H_67_N_2_O_10_P_2_ (M+H)^+^: 1061.4271, found: 1061.4294.

*1,6-Dideoxy-1,6-dimethansulfonamide-2,3,4,5-tetra-O-benzyl Mannitol (* ***10***). Compound** 8** (117 mg, 0.20 mmol) was dissolved in dry toluene (4 mL) and PPh_3_ on polystyrene (280 mg, 3.1 mmol/g, 0.87 mmol) was added and the mixture was stirred at 60°C for 18 h. The mixture was allowed to cool and mesyl chloride (0.10 mL, 1.29 mmol) was added. The mixture was stirred at r.t. under N_2_ for 90 min and then Na_2_CO_3_ (3 mL, sat. aq.) was added. This mixture was stirred at r.t. for an additional 18 h before the solid support was filtered and washed with EtOAc. The organic phase was washed twice with H_2_O and the aqueous phase was extracted once with EtOAc. The combined organic phase was dried (MgSO_4_) and concentrated. The residue was purified by chromatography (Biotage Isolera One, SNAP 25 g, EtOAc 5–95% in heptane) to give** 10** (774 mg, 60%) as a clear oil. [*α*]_D_^23^ +26 (c 0.9, C_6_H_6_), ^1^H NMR (C_6_D_6_): *δ* 7.41–7.43 (m, 4H, ArH), 7.27–7.29 (m, 4H, ArH) 7.15–7.22 (m, 8H, ArH), 7.05–7.12 (m, 4H, ArH), 4.80, 4.71 (ABq, 2H each,* J* 11.3 Hz, PhCH_2_), 4.60 (t, 2H,* J* 6.2 Hz, NH), 4.42, 4.38 (ABq, 2H each,* J* 11.6 Hz, PhCH_2_), 3.92–3.93 (m, 2H, H-3, H-4), 3.74–3.79 (m, 2H, H-2, H-5), 3.26–3.39 (m, 4H, H-1, H-1′, H-6, H-6′), 2.26 (s, 6H, SCH_3_). ^13^C NMR (C_6_D_6_): *δ* 138.9, 138.4, 128.9, 128.8, 128.6, 128.4, 79.9, 79.5, 74.8, 72.4, 43.2, 39.6. HRMS (ESI) calcd. for C_36_H_45_N_2_O_8_S_2_ (M+H)^+^: 697.2617, found: 697.2624.

### 2.2. Microbiology

*E. coli* DH5*α* and* Pseudomonas putida* were precultured overnight in shake flasks containing lysogeny broth (LB) media (10 g/L Bacto tryptone, 5 g/L Bacto yeast extract, and 10 g/L NaCl, pH 7.0) in an orbital shake incubator set to 180 rpm and 37°C for* E. coli* or 30°C for* P. putida*. The bacteria were transferred to an LB-agar plate and incubated at r.t. for 30 min. Compounds** 3**,** 4** and Novobiocin were dissolved in sterile water to a concentration of 10 mg/mL and transferred to blank discs (Oxoid) according to Table 1. Polymyxin B, 300 units (Oxoid CT0044), and Tetracycline 30 mg (Oxoid CT0054) disc were purchased from Oxoid. The discs were allowed to dry for 5 min before they were transferred to the LB-agar plate and incubated at r.t. for 1 h followed by incubation overnight at 37°C for* E. coli* and 30°C for* P. putida*. All experiments were run in duplicate.

## 3. Results and Discussion

**Figure 3:**
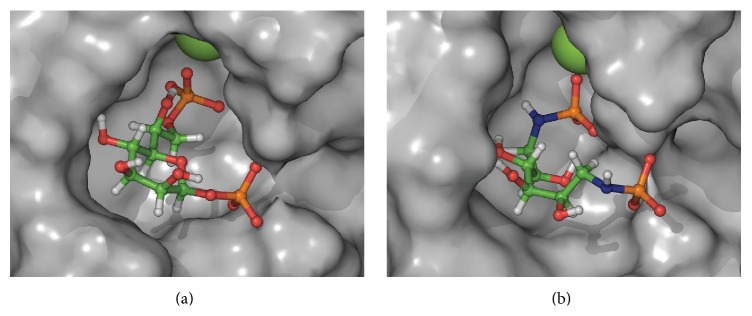
(a) d-Glycero-*β*-d-manno-heptose 1,7-bisphosphate (**1**) in the binding pocket of GmhB according to the crystal structure from [23]. (b) Molecular dynamic snapshot of 1,6-dideoxy-1,6-diphosphoramidate mannitol (**3**) in the binding pocket of GmhB.

Molecular dynamics simulations of the two proposed structures were performed on a model based on the X-ray diffraction data of GmhB enzyme from* E. coli*, with its natural substrate (PDB 3L8G) [23]. The model was prepared using the Schrödinger 2012 software suite by application of the protein preparation tool, followed by energy optimization utilizing the OPLS-2005 forcefield and the GB/SA solvation method for water. For each of the new ligands, a MCMM conformational search utilizing the OPLS-2005 forcefield and the GB/SA solvation method for water was performed. The low energy conformer most similar to the natural ligand was placed on the protein by superimposition, followed by a 2.4 ns molecular dynamics simulation of an orthorhombic box with 10 Å buffer of water molecules, using the default settings of Desmond 2012 [24, 25]. The dimethylsulfonamide** 4** did not retain in the binding pocket, perhaps due to the poor interaction with the magnesium ion, and was not a potential inhibitor according to the docking study. The phosphoramidate** 3** on the other hand retained in the binding pocket; this makes it an interesting compound to evaluate in a biological assay. The natural substrate** 1**, which also retained in the binding pocket, and a snapshot from the molecular dynamics of** 3** are displayed in Figure 3 [26]. Despite the poor interactions of** 4** in the binding pocket we decided to synthesize and evaluate both analogs in an effort to better understand the system.

**Scheme 1:**
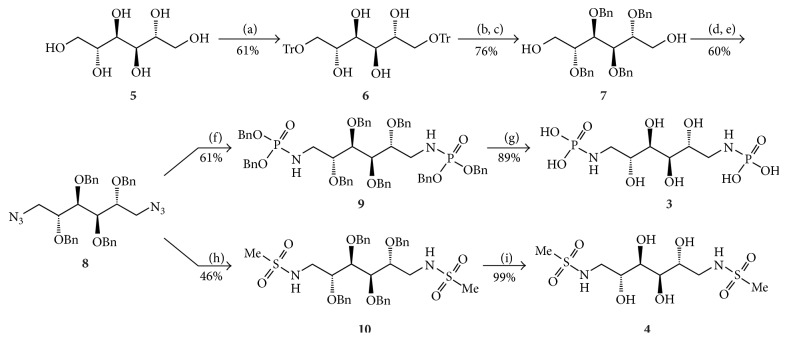
Reagents and conditions: (a) Ph_3_CCl, AgNO_3_, pyridine, r.t.; (b) BnBr, NaH, THF, r.t.; (c) TFA, n-BuOH, CH_2_Cl_2_, r.t.; (d) TsCl, pyridine, r.t.; (e) NaN_3_, DMSO, 100–150°C; (f) P(OBn)_3_, toluene, r.t. to 90°C; (g) Pd/C, H_2_, EtOAc/EtOH/H_2_O, r.t.; (h) MsCl, PS-PPh_3_, toluene, r.t. to 60°C; and (i) Pd/C, H_2_, EtOAc/EtOH/H_2_O r.t.

Mannitol (**5**) was selectively ditritylated in positions 1 and 6 using trityl chloride to give 1,6-di-*O*-trityl mannitol (**6**) (Scheme 1) in 76% yield. The tritylated product** 6** was then benzylated followed by detritylation using TFA to get 2,3,4,5-tetra-*O*-benzyl mannitol (**7**) in 76% according to a previously described method [19]. Compound** 7** was tosylated with 4-toluenesulfonyl chloride followed by installation of an azide to give 1,6-azido-1,6-dideoxy-2,3,4,5-tetra-*O*-benzyl mannitol (**8**) in 60% yield over two steps. The azide** 8** reacted with tribenzyl phosphite in a Staudinger type reaction overnight to yield 1,6-dideoxy-1,6-di-(di-benzyl-phosphoramidate)-2,3,4,5-tetra-*O*-benzyl mannitol (**9**) in 61% yield [22, 27].

The formation of tribenzyl phosphite from phosphorous chloride and benzyl alcohol was troublesome and very sensitive to moisture and air. The reagents had to be freshly distilled prior to the reaction and filtered, to remove triethylammonium chloride, under a nitrogen atmosphere to eliminate the formation of tribenzyl phosphate. Compound** 9** was then hydrogenolysed in ethyl acetate/ethanol/water (3 : 5 : 2) in the presence of Pd/C to give 1,6-dideoxy-1,6-diphosphoramidate mannitol (**3**) in 89% yield [27].

To synthesize the dimethylsulfonamide analog, compound** 8** was treated under Staudinger conditions with triphenylphosphine on solid support for 18 h at 60°C followed by addition of methanesulfonyl chloride to form 1,6-dideoxy-1,6-dimethansulfonamide-2,3,4,5-tetra-*O*-benzyl mannitol (**10**) in 46% yield (Scheme 1) [27]. Compound** 10** was then hydrogenolysed under the same conditions as** 9**, but more Pd/C had to be added since sulfur residue poisoned the catalyst. This gave 1,6-dideoxy-1,6-dimethansulfonamide mannitol (**4**) in 99% yield.

To evaluate the biological properties of these compounds, the cell growth was monitored for two different Gram-negative bacteria,* E. coli* and* P. putida*, in the Kirby-Bauer antibiotic susceptibility test with and without addition of Novobiocin. Complete loss of access to l,d-Hep would result in bacterial cells with a truncated LPS. The impairment would result in hypersensitivity towards a range of compounds including the antibiotic Novobiocin that is usually not effective against Gram-negative bacteria [11]. Discs were impregnated with** 3**,** 4** or a mixture of Novobiocin and** 3** or** 4** according to Table 1 and placed on a LB-agar plate that had been streaked with the bacteria. Positive controls (Tetracycline and Polymyxin B) and water as a negative control were also added and the plates were incubated overnight at 37°C for* E. coli* and 30°C for* P. putida*.

Only Tetracycline and Polymyxin B (Table 1, entries 8-9) showed inhibition of cell growth for both* E. coli* and* P. putida* and no inhibition was seen for** 3**,** 4**, Novobiocin, or Novobiocin in combination with** 3** or** 4** (Table 1, entries 1–7). The absence of activity in the Kirby-Bauer antibiotic susceptibility test could be contingent on several different things; for example, the compounds might not be competitive inhibitors for the enzyme, or other mechanisms such as uptake or efflux may be responsible for the lack of activity.

## 4. Conclusion

To summarize, we have designed, synthesized, and evaluated two new mannitol-based compounds aimed at inhibiting the enzyme GmhB in the l,d-Hep biosynthesis in Gram-negative bacteria. All levels of the l,d-Hep biosynthesis are promising and underexplored targets for new antibiotics and GmhB is an attractive enzyme to target. A deletion of the gene that codes for GmhB in* E. coli* does not give a completely heptoseless LPS, indicating that there are other enzymes that will partly compensate for this protein [13]. However, in other bacteria, the protein GmhB is crucial [28] for the production of l,d-Hep, something that can be utilized in the design of more specific antibiotic agents. The compounds were evaluated* in silico* and in the Kirby-Bauer antibiotic susceptibility test on two different Gram-negative bacteria. While 1,6-dideoxy-1,6-diphosphoramidate mannitol (**3**) showed encouraging results* in silico*, neither of them showed any activity in the Kirby-Bauer test. However, the encouraging result* in silico* merits further investigation into mannitol-based inhibitors for GmhB, something that is currently ongoing in our laboratory.

## Supplementary Material

Proton and carbon NMR spectra of novel compounds are provided in the Supplementary Material.

## Figures and Tables

**Table 1:** Substances tested in duplicate on *E. coli* and *P. putida*.

Entry	Substance	Amount	Inhibited cell growth
1	**3**	100 mg	No
2	**3** + Novobiocin	100 mg + 50 mg	No
3	**3** + Novobiocin	50 mg + 50 mg	No
4	**4**	100 mg	No
5	**4** + Novobiocin	100 mg + 50 mg	No
6	**4** + Novobiocin	50 mg + 50 mg	No
7	Novobiocin	50 mg	No
8	Polymyxin B^a^	300 units	Yes
9	Tetracycline^a^	30 mg	Yes
10	H_2_O	10 mg	No

^a^Discs pretreated with antibiotic from Oxoid.
