# Supplementary material for: Synthesis and Evaluation of Mannitol-Based Inhibitors for Lipopolysaccharide Biosynthesis
Source: Int J Med Chem. 2016 Feb 11;2016:3475235. doi: 10.1155/2016/3475235 (PMC4766332; doi:10.1155/2016/3475235)

NAME RJ–133b
EXPNO 1
PROCNO 1
Date_ 20120705
Time 10.13
INSTRUM spect
PROBHD 5 mm PABBO BB/
PULPROG zg30
TD 57690
SOLVENT C6D6
NS 64
DS 2
SWH 7211.539 Hz
FIDRES 0.125005 Hz
AQ 3.9998901 sec
RG 256
DW 69.333 usec
DE 6.00 usec
TE 298.4 K
D1 0.00100000 sec
TD0 1

======= CHANNEL f1 =======
NUC1 1H
P1 10.00 usec
PL1 –4.00 dB
PL1W 21.45254898 W
SFO1 400.1334011 MHz
SI 131072
SF 400.1300447 MHz
WDW EM
SSB 0
LB 0.30 Hz
GB 0
PC 4.00

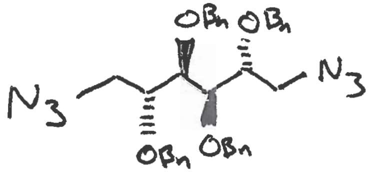

1 H-NMR of Compound 8

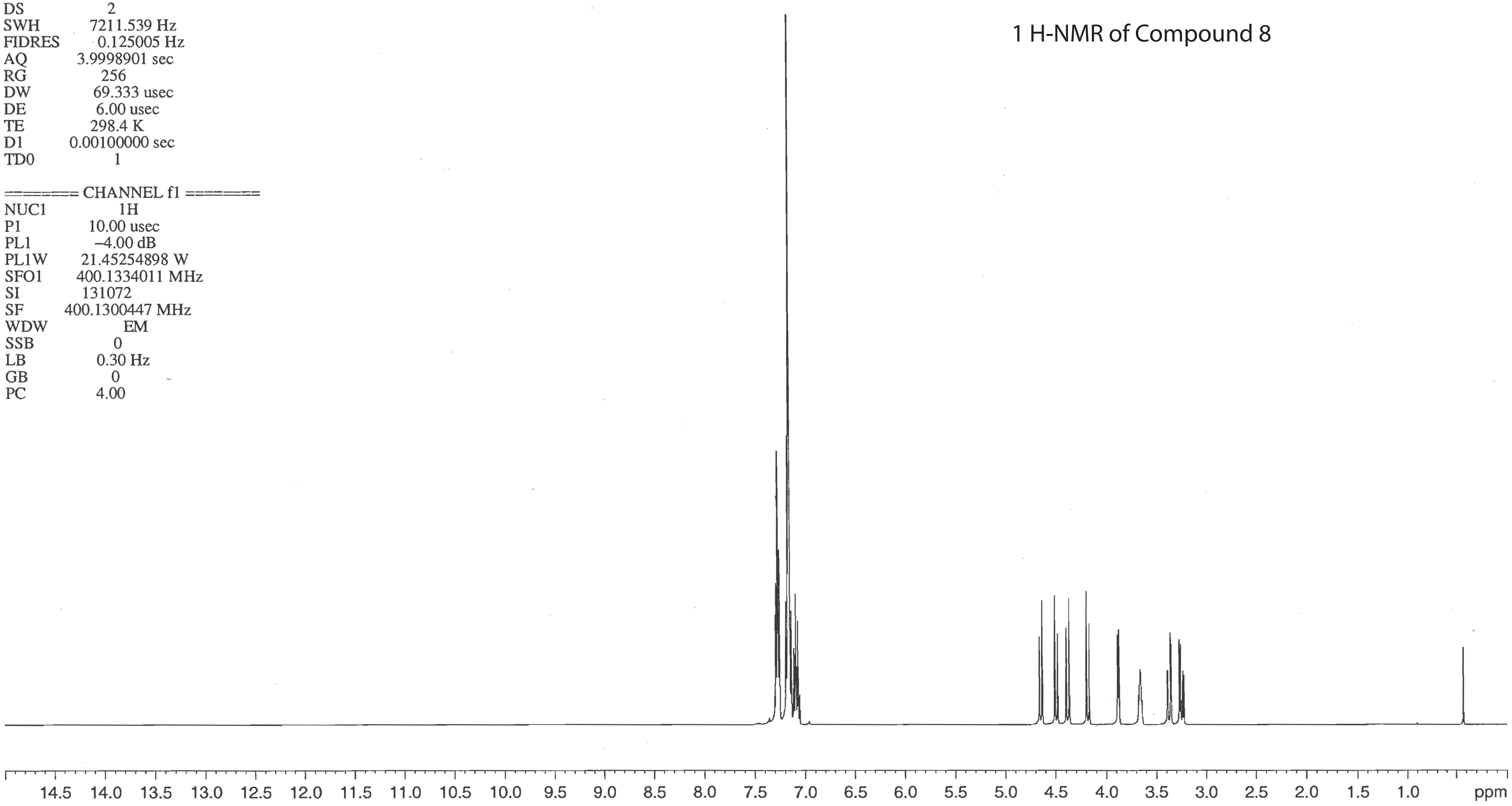

NAME	RJ–133b
EXPNO	3
PROCNO	1
Date_	20120705
Time	23.50
INSTRUM	spect
PROBHD	5 mm PABBO BB/
PULPROG	zgdc30
TD	48074
SOLVENT	C6D6
NS	8000
DS	4
SWH	24038.461 Hz
FIDRES	0.500030 Hz
AQ	0.9999892 sec
RG	57
DW	20.800 usec
DE	6.00 usec
TE	300.5 K
D1	0.00100000 sec
D11	0.03000000 sec
TD0	1

======= CHANNEL f1 =======

NUC1	13C
P1	8.50 usec
PL1	–3.00 dB
PL1W	58.63890457 W
SFO1	100.6228298 MHz

======= CHANNEL f2 =======

CPDPRG2	waltz16
NUC2	1H
PCPD2	80.00 usec
PL2	–4.00 dB
PL12	14.06 dB
PL2W	21.45254898 W
PL12W	0.33533499 W
SFO2	400.1316005 MHz
SI	32768
SF	100.6127429 MHz

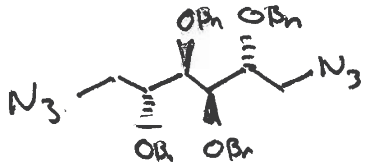

13 C-NMR of Compound 8

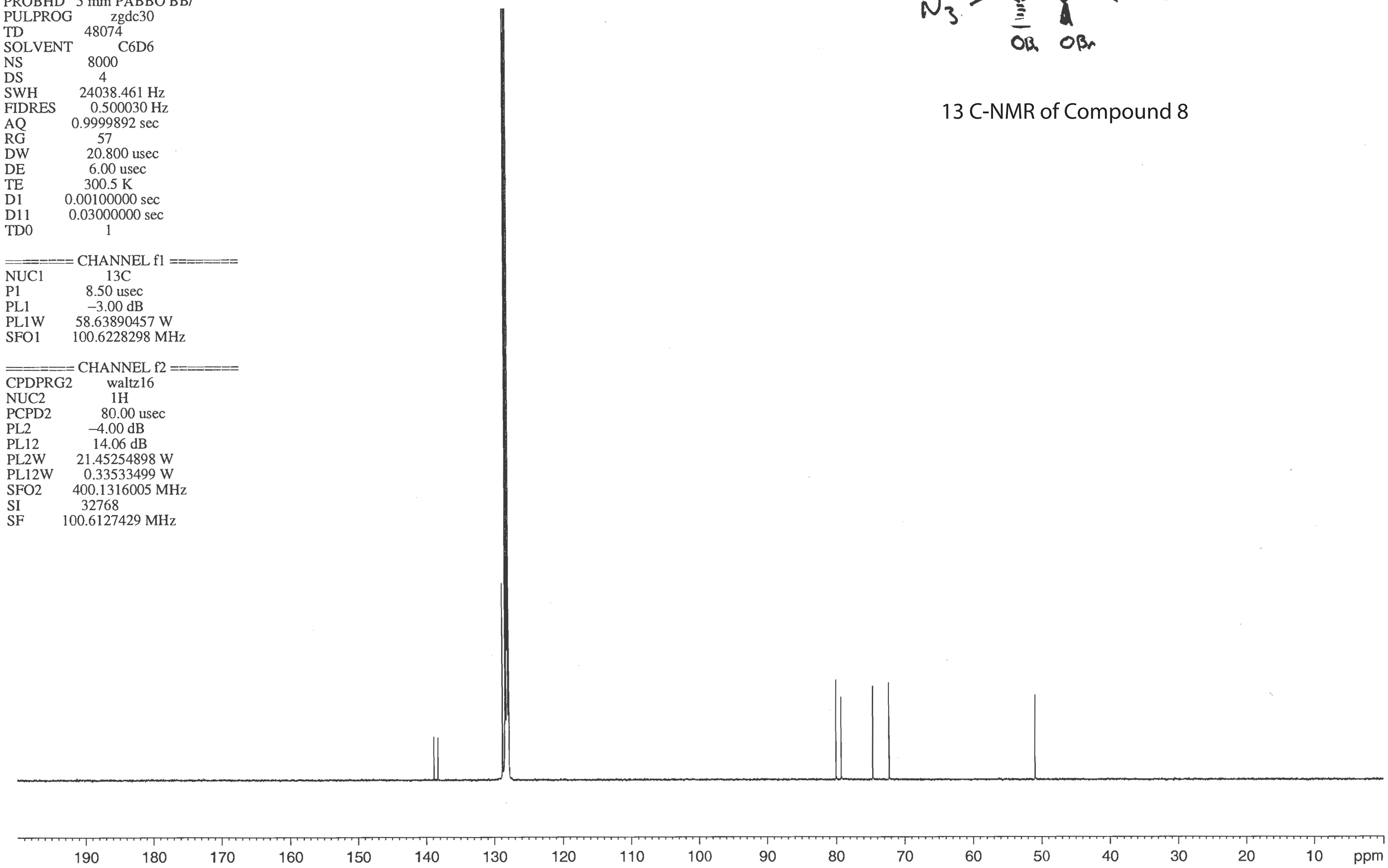

NAME RJ–183c
EXPNO 1
PROCNO 1
Date_ 20121010
Time 15.11
INSTRUM spect
PROBHD 5 mm PABBO BB/
PULPROG zg30
TD 57690
SOLVENT C6D6
NS 128
DS 2
SWH 7211.539 Hz
FIDRES 0.125005 Hz
AQ 3.9998901 sec
RG 406
DW 69.333 usec
DE 6.00 usec
TE 294.4 K
D1 0.00100000 sec
TD0 1

======= CHANNEL f1 =======
NUC1 1H
P1 10.00 usec
PL1 –4.00 dB
PL1W 21.45254898 W
SFO1 400.1334011 MHz
SI 131072
SF 400.1300447 MHz
WDW EM
SSB 0
LB 0.30 Hz
GB 0
PC 4.00

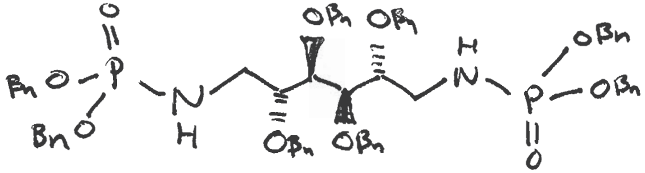

1 H-NMR of Compound 9

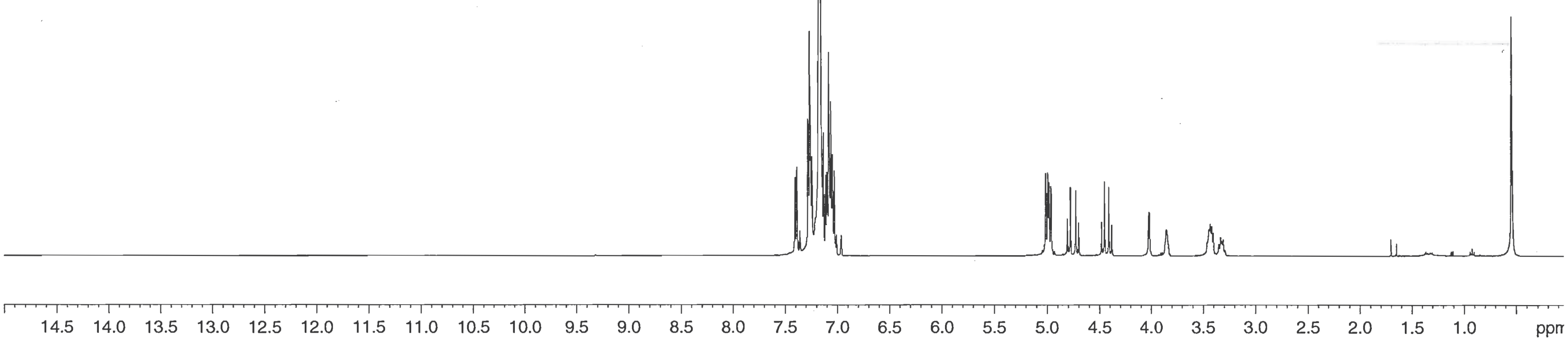

NAME RJ-183c
EXPNO 3
PROCNO 1
Date_ 20121011
Time 4.59
INSTRUM spect
PROBHD 5 mm PABBO BB/
PULPROG zgdc30
TD 48074
SOLVENT C6D6
NS 10000
DS 4
SWH 24038.461 Hz
FIDRES 0.500030 Hz
AQ 0.9999892 sec
RG 57
DW 20.800 usec
DE 6.00 usec
TE 295.9 K
D1 0.00100000 sec
D11 0.03000000 sec
TD0 1

======== CHANNEL f1 ========
NUC1 13C
P1 8.50 usec
PL1 -3.00 dB
PL1W 58.63890457 W
SFO1 100.6228298 MHz

======== CHANNEL f2 ========
CPDPRG2 waltz16
NUC2 1H
PCPD2 80.00 usec
PL2 -4.00 dB
PL12 14.06 dB
PL2W 21.45254898 W
PL12W 0.33533499 W
SFO2 400.1316005 MHz
SI 32768
SF 100.6127437 MHz

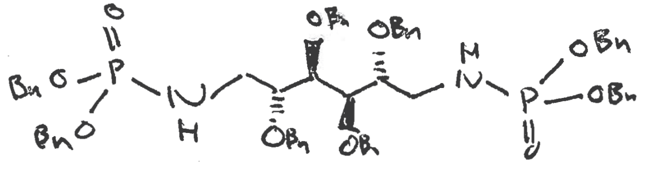

13 C-NMR of Compound 9

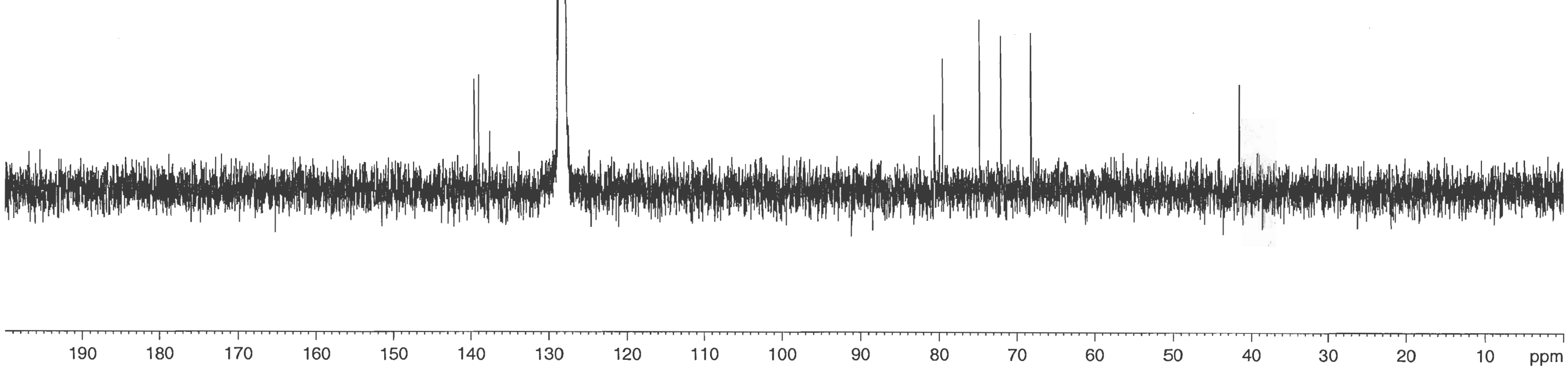

NAME	RJ–197b
EXPNO	1
PROCNO	1
Date_	20121120
Time	9.31
INSTRUM	spect
PROBHD	5 mm PABBO BB/
PULPROG	zg30
TD	57690
SOLVENT	D2O
NS	128
DS	2
SWH	7211.539 Hz
FIDRES	0.125005 Hz
AQ	3.9998901 sec
RG	362
DW	69.333 usec
DE	6.00 usec
TE	294.5 K
D1	0.00100000 sec
TD0	1

======= CHANNEL f1 =======

NUC1	1H
P1	10.00 usec
PL1	–4.00 dB
PL1W	21.45254898 W
SFO1	400.1334011 MHz
SI	131072
SF	400.1299643 MHz
WDW	EM
SSB	0
LB	0.30 Hz
GB	0
PC	4.00

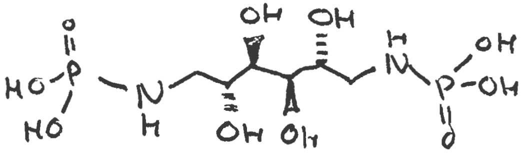

1 H-NMR of Compound 3

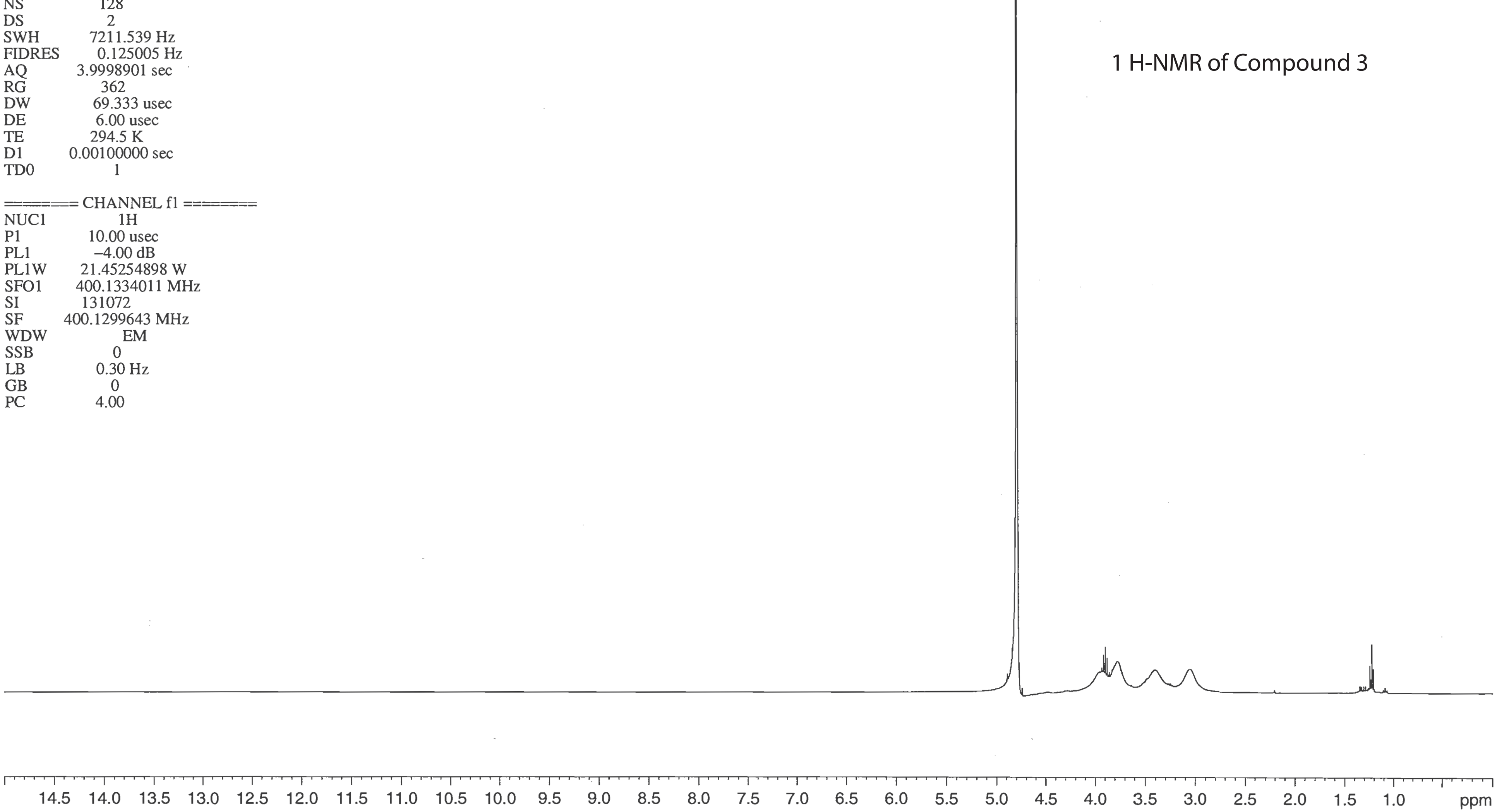

NAME RJ–197b
EXPNO 3
PROCNO 1
Date_ 20121121
Time 0.56
INSTRUM spect
PROBHD 5 mm PABBO BB/
PULPROG zgdc30
TD 48074
SOLVENT D2O
NS 10000
DS 4
SWH 24038.461 Hz
FIDRES 0.500030 Hz
AQ 0.9999892 sec
RG 57
DW 20.800 usec
DE 6.00 usec
TE 296.0 K
D1 0.00100000 sec
D11 0.03000000 sec
TD0 1

======= CHANNEL f1 =======
NUC1 13C
P1 8.50 usec
PL1 –3.00 dB
PL1W 58.63890457 W
SFO1 100.6228298 MHz

======= CHANNEL f2 =======
CPDPRG2 waltz16
NUC2 1H
PCPD2 80.00 usec
PL2 –4.00 dB
PL12 14.06 dB
PL2W 21.45254898 W
PL12W 0.33533499 W
SFO2 400.1316005 MHz
SI 32768
SF 100.6127690 MHz

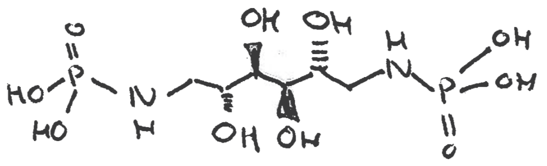

13 C-NMR of Compound 3

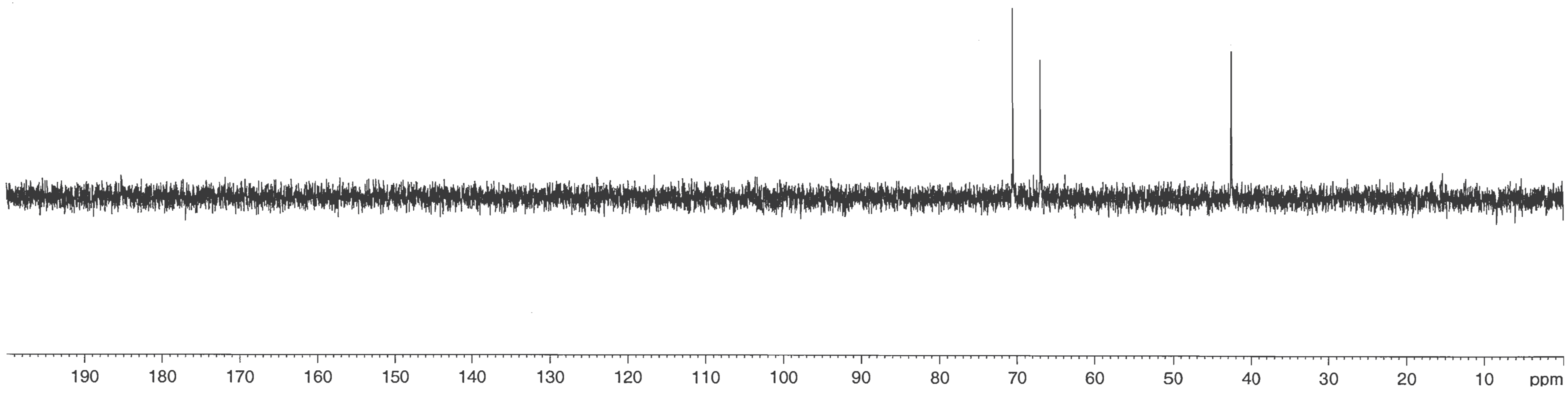

NAME RJ–149b
EXPNO 1
PROCNO 1
Date_ 20120723
Time 17.14
INSTRUM spect
PROBHD 5 mm PABBO BB/
PULPROG zg30
TD 57690
SOLVENT C6D6
NS 32
DS 2
SWH 7211.539 Hz
FIDRES 0.125005 Hz
AQ 3.9998901 sec
RG 228
DW 69.333 usec
DE 6.00 usec
TE 298.6 K
D1 0.00100000 sec
TD0 1

======= CHANNEL f1 ========
NUC1 1H
P1 10.00 usec
PL1 –4.00 dB
PL1W 21.45254898 W
SFO1 400.1334011 MHz
SI 131072
SF 400.1300431 MHz
WDW EM
SSB 0
LB 0.30 Hz
GB 0
PC 4.00

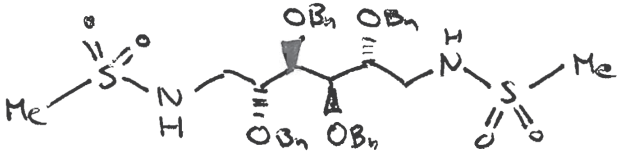

1 H-NMR of Compound 10

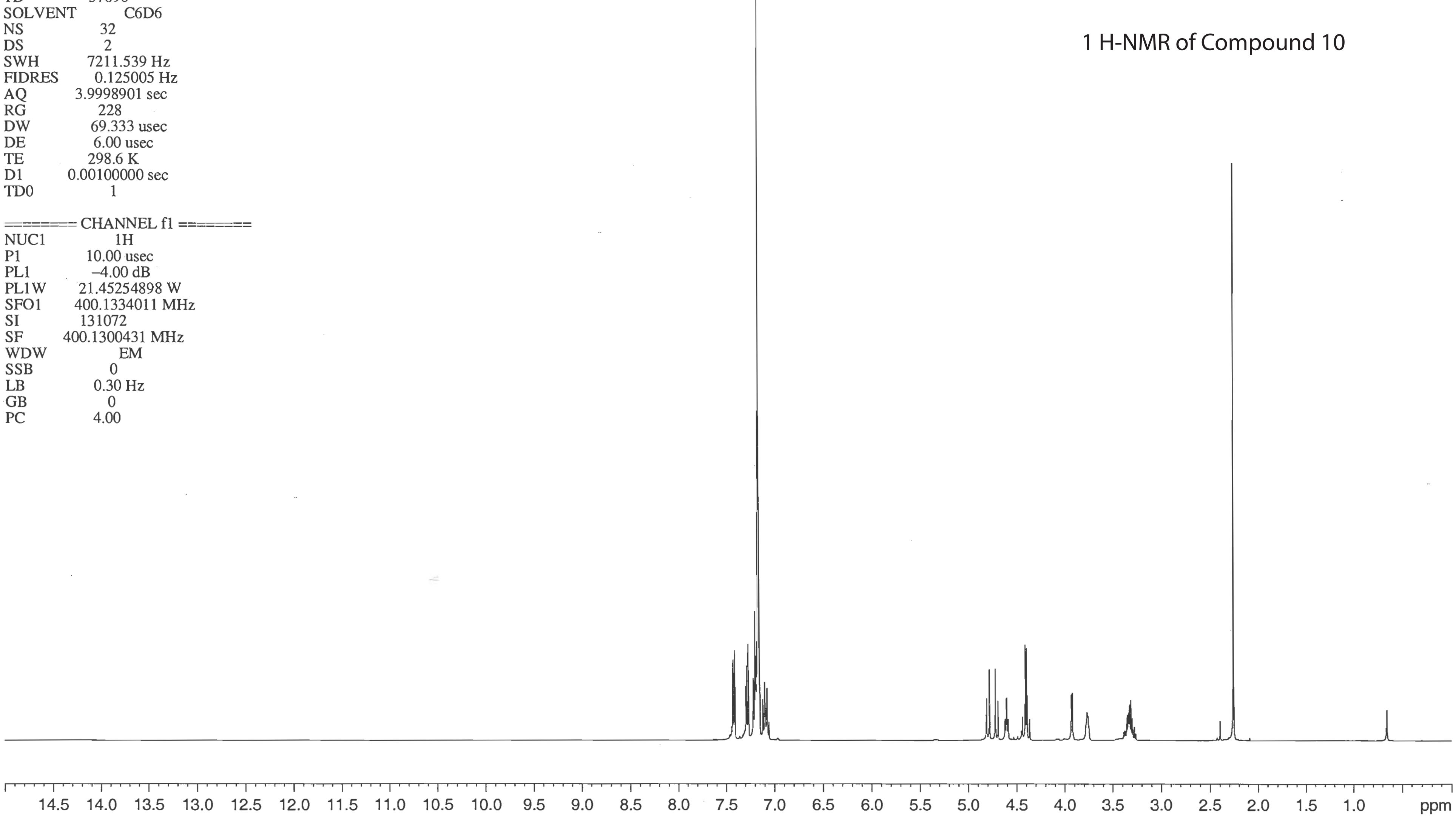

NAME RJ–149b
EXPNO 3
PROCNO 1
Date_ 20120723
Time 23.06
INSTRUM spect
PROBHD 5 mm PABBO BB/
PULPROG zgdc30
TD 48074
SOLVENT C6D6
NS 9000
DS 4
SWH 24038.461 Hz
FIDRES 0.500030 Hz
AQ 0.9999892 sec
RG 57
DW 20.800 usec
DE 6.00 usec
TE 300.1 K
D1 0.00100000 sec
D11 0.03000000 sec
TD0 1

======== CHANNEL f1 ========
NUC1 13C
P1 8.50 usec
PL1 –3.00 dB
PL1W 58.63890457 W
SFO1 100.6228298 MHz

======== CHANNEL f2 ========
CPDPRG2 waltz16
NUC2 1H
PCPD2 80.00 usec
PL2 –4.00 dB
PL12 14.06 dB
PL2W 21.45254898 W
PL12W 0.33533499 W
SFO2 400.1316005 MHz
SI 32768
SF 100.6127429 MHz

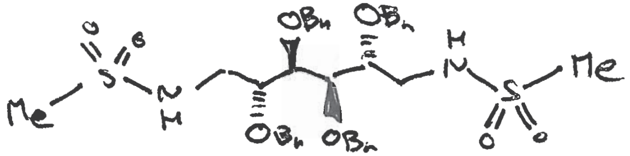

13 C-NMR of Compound 10

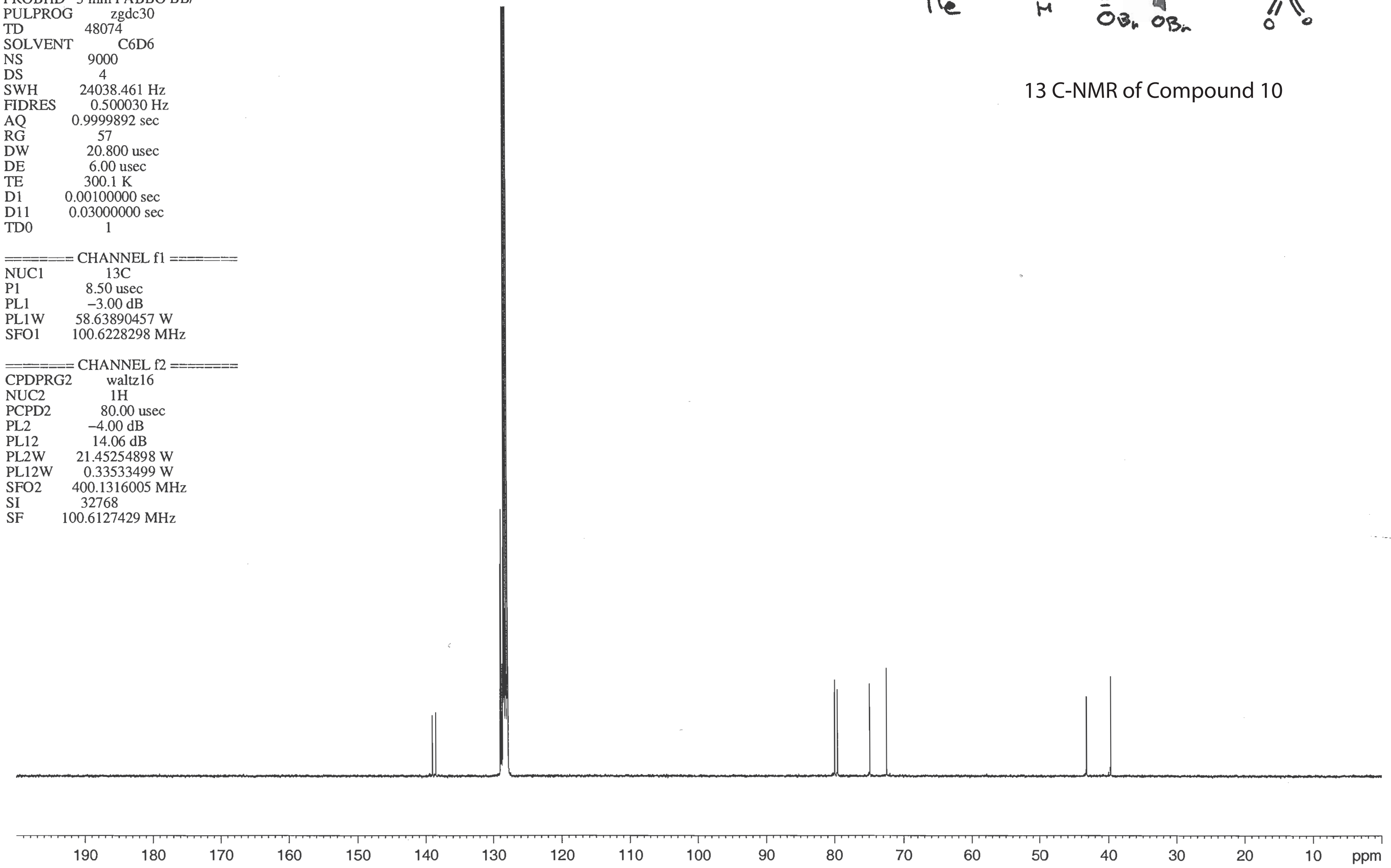

NAME RJ–195a
EXPNO 1
PROCNO 1
Date_ 20121029
Time 10.42
INSTRUM spect
PROBHD 5 mm PABBO BB/
PULPROG zg30
TD 57690
SOLVENT D2O
NS 64
DS 2
SWH 7211.539 Hz
FIDRES 0.125005 Hz
AQ 3.9998901 sec
RG 406
DW 69.333 usec
DE 6.00 usec
TE 294.4 K
D1 0.00100000 sec
TD0 1

======= CHANNEL f1 =======
NUC1 1H
P1 10.00 usec
PL1 –4.00 dB
PL1W 21.45254898 W
SFO1 400.1334011 MHz
SI 131072
SF 400.1299637 MHz
WDW EM
SSB 0
LB 0.30 Hz
GB 0
PC 4.00

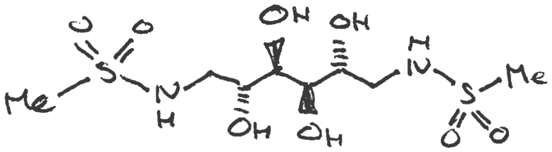

1 H-NMR of Compound 4

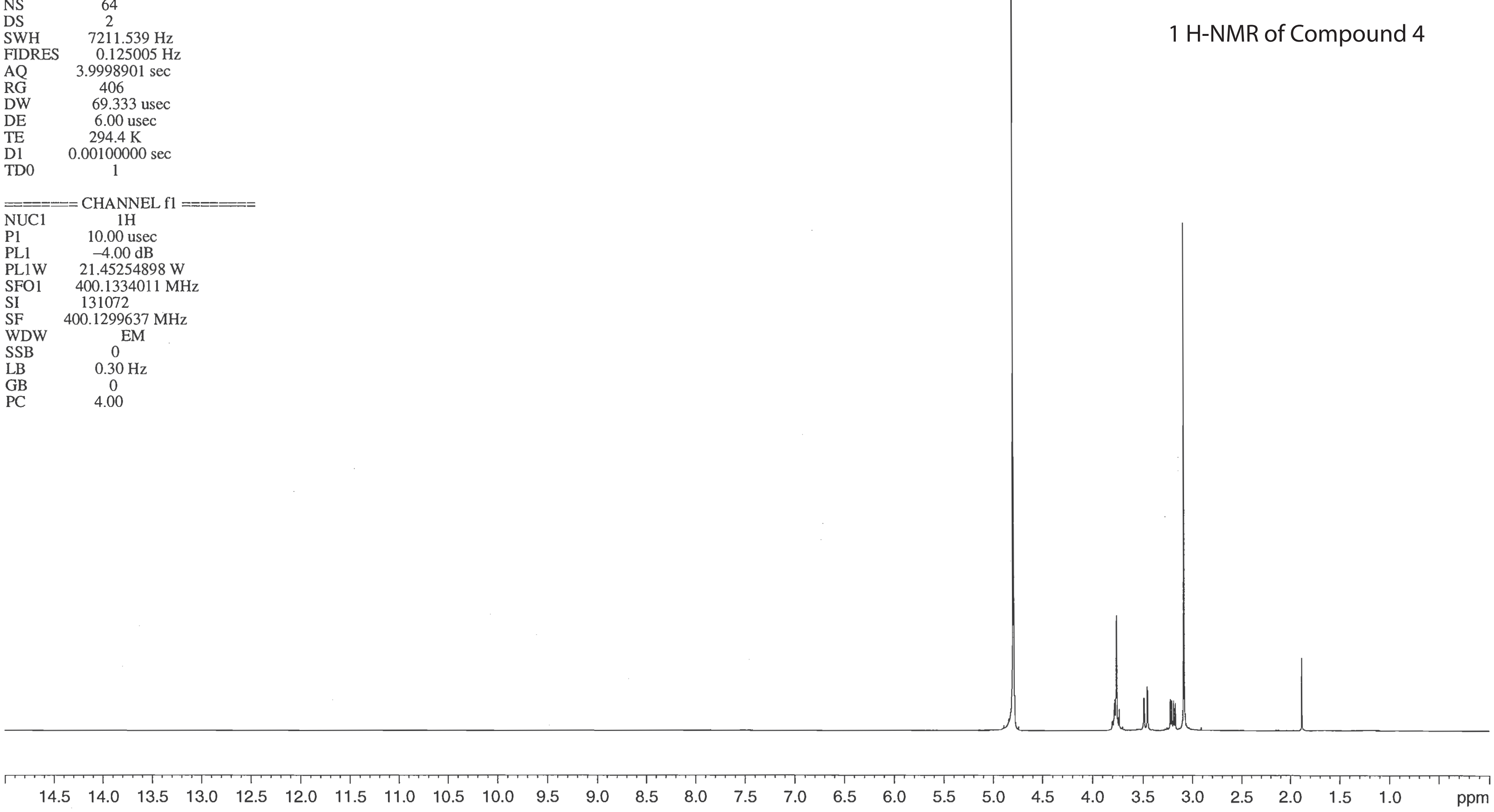

NAME	RJ–195a
EXPNO	4
PROCNO	1
Date_	20121030
Time	5.11
INSTRUM	spect
PROBHD	5 mm PABBO BB/
PULPROG	zgdc30
TD	48074
SOLVENT	D2O
NS	10000
DS	4
SWH	24038.461 Hz
FIDRES	0.500030 Hz
AQ	0.9999892 sec
RG	57
DW	20.800 usec
DE	6.00 usec
TE	296.0 K
D1	0.00100000 sec
D11	0.03000000 sec
TD0	1
======= CHANNEL f1 =======	
NUC1	13C
P1	8.50 usec
PL1	–3.00 dB
PL1W	58.63890457 W
SFO1	100.6228298 MHz
======= CHANNEL f2 =======	
CPDPRG2	waltz16
NUC2	1H
PCPD2	80.00 usec
PL2	–4.00 dB
PL12	14.06 dB
PL2W	21.45254898 W
PL12W	0.33533499 W
SFO2	400.1316005 MHz
SI	32768
SF	100.6127690 MHz

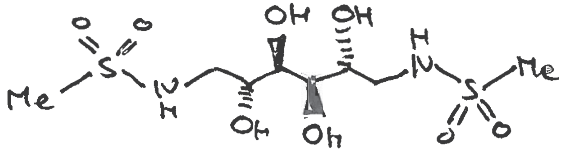

13 C-NMR of Compound 4

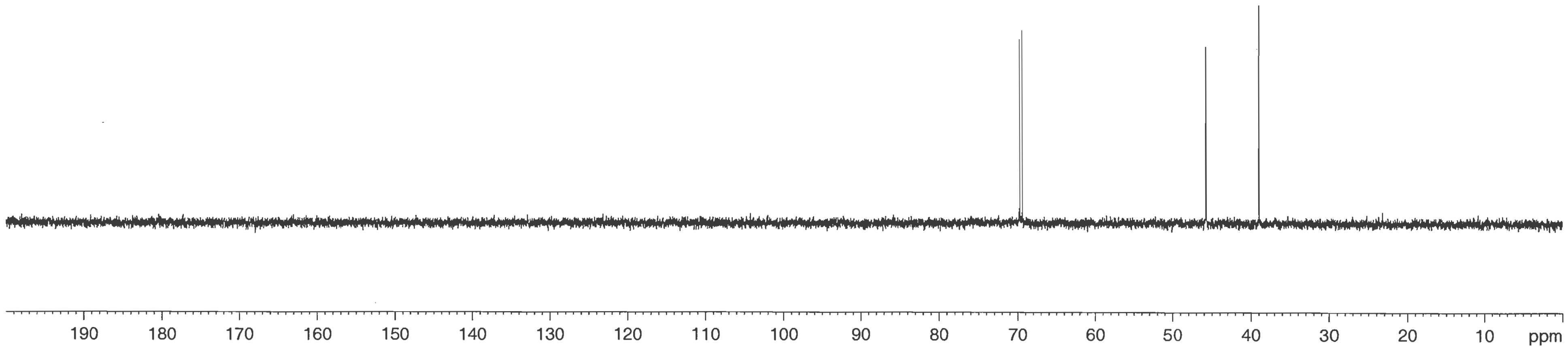

Supplement: Supplementary file 1 — Proton and carbon NMR spectra of novel compounds are provided in the Supplementary Material. [file 3475235.f1.pdf]
